# Shame in medical clerkship: “You just feel like dirt under someone’s shoe”

**DOI:** 10.1007/s40037-021-00665-w

**Published:** 2021-05-05

**Authors:** Beth Whelan, Stefan Hjörleifsson, Edvin Schei

**Affiliations:** 1 Student Health and Wellness Center, Memorial University, St. John’s, NL Canada; 2 Department of Global Public Health and Primary Care, University of Bergen, Bergen, Norway; 3 Centre for Medical Education and Department of Global Public Health and Primary Care, University of Bergen, Bergen, Norway

**Keywords:** Undergraduate medical students, Shame, Learning environment, Impact on learning, Burnout

## Abstract

**Introduction:**

This study explores how senior medical students’ experience and react to shame during clinical placements by asking them to reflect on (1) manifestations of shame experiences, (2) situations and social interactions that give rise to shame, and (3) perceived effects of shame on learning and professional identity development.

**Methods:**

In this interpretive study, the authors recruited 16 senior medical students from two classes at a Norwegian medical school. In three focus group interviews, participants were invited to reflect on their experiences of shame. The data were analyzed using *systematic text condensation*, producing rich descriptions about students’ shame experiences.

**Results:**

All participants had a range of shame experiences, with strong emotional, physical, and cognitive reactions. Shame was triggered by a range of clinician behaviours interpreted as disinterest, disrespect, humiliation, or breaches of professionalism. Shame during clinical training caused loss of confidence and motivation, worries about professional competence, lack of engagement in learning, and distancing from shame-associated specialties. No positive effects of shame were reported.

**Discussion:**

Shame reactions in medical students were triggered by clinician behaviour that left students feeling unwanted, rejected, or burdensome, and by humiliating teaching situations. Shame had deleterious effects on motivation, learning, and professional identity development. This study has implications for learners, educators, and clinicians, and it may contribute to increased understanding of the importance of supportive learning environments and supervisors’ social skills within the context of medical education.

## Introduction

Shame is a powerful, deeply uncomfortable, self-denigrating emotion that influences behaviour and identity by sensitizing individuals to the perceived opinions of others [1–5]. Shame inheres in the experience of seeing oneself as critically flawed in relation to some internalized ideal, thereby judging one’s self as globally deficient or unworthy [3, 6].

Shame plays an important role in the development of normal social behaviour and moral conscience [1, 5]. In complex social settings, such as clinical learning environments, the universal desire to avoid shame fuels socialization, through nonconscious conformity with the explicit and tacit norms of the environment [7–9].

While shame can benefit social integration, strategies to avoid shame can cause substantial distress and maladaptive behaviours [10]. Medical learners who experience shame are more likely to withdraw from others, hide, deny responsibility, ignore problems, and respond with anger [4, 11]. In a survey of surgical trainees, 70% of respondents experienced shame as a result of derogatory behavior, such as being called “stupid” or threatened, most often by supervisors and senior colleagues. Medical residents who had been shamed were more likely to experience severe burnout symptoms, including depersonalization, depression, isolation, and poor job performance [12].

In clerkship, medical students are in a particularly vulnerable position, as peripheral and relatively incompetent participants expected to quickly learn and adapt to unspecified standards of behavior and performance, in unfamiliar work environments characterized by high-stake, specialized activities, time pressure, hierarchy, and emotionally charged interactions with suffering patients [13]. The students’ grades and future careers depend on their superiors’ appraisals, while professional learning and emotional health hinge on guidance and adequate performance feedback from supervisors and role models [14–16]. As shown by Dornan, affective support is the most crucial element in successful clinical learning and professional identity development [17, 18].

Shame during clinical rotations could impact negatively on medical students’ learning, well-being, and professional identity development [19, 20], as indicated by studies stating that students are frequently subject to subtle mistreatment [21] and teaching by belittlement [22].

The literature addressing medical students’ experiences of shame in clerkship, and how shame events impact learning, is in a nascent state, and few studies have been carried out, mostly in the US [4, 11]. Thus, in this Scandinavian study, we asked senior medical students to reflect on (1) the manifestations of shame experiences during clerkship, (2) the situations and social interactions that give rise to shame, and (3) the perceived consequences of those shame reactions on learning and professional identity development.

## Material and methods

We conducted the study from an interpretive and critical realist epistemological stance, assuming firstly that in scientific knowledge production the researchers’ perspectives and the perspectives of their research participants inevitably influence the results, and secondly that investigating people’s experiences in the social world allows for a gradual understanding of underlying psychological and social structures and mechanisms [23]. In order to leverage the sharing, commenting, and relating that occurs during discussion, we collected data from focus group interviews with senior medical students in Norway. The transcribed interviews were analyzed using *systematic text condensation*, a method for thematic cross-case analysis [24].

### Context

In Norway, undergraduate medical education comprises six years, with an emphasis on clinical training during the last three years. Clinical practice at local hospitals takes place in three 8‑week periods with frequent rotations between departments. In teaching hospitals, all physicians, including residents, are expected to supervise and support medical students during their placements. However, most supervisors lack formal pedagogical training.

### Participants and recruitment

Students from years 5 and 6 were recruited using a snowball technique. Class representatives were informed about the study topic, were told that the group interviews were being conducted in English, and were asked to forward the invitation to classmates through direct contact and social media. Interested students were asked to contact BW directly by email to confirm participation. No incentives, apart from coffee and snacks, were offered. BW and ES conducted three 120-minute focus group interviews with sixteen participants. Twelve students were female, which represents the ratio of female to male students in the University of Bergen medical school; age ranged from 24–33 years. Each group contained students from the same class, with two groups of fifth-year and one group of sixth-year students. To strengthen reflexivity, the authors identified sources of bias before and after each interview. Strengthening psychological safety in the groups was deemed essential [25]. Researcher ES was familiar to the students as a teacher and proponent of medical professionalism but was not involved in their current or subsequent courses. Researcher BW was a visiting psychology professor and was not familiar with the students in any context.

### Data collection

In an introductory email, we asked participants to think about medical education experiences that they associated with feelings of shame. We included this prompt so that participants would reflect on experiences of shame prior to the focus group. We did not provide a definition of shame in the email, as we wanted students to independently explore all events, thoughts, and feelings that they thought were shame related. At the beginning of the interview, participants were provided with informed consent and confidentiality forms. BW and ES moderated the interviews using a semi-structured interview guide. Participants were invited to share experiences they had reflected on in advance, or any other experiences of shame in the context of their medical education. Students described in detail, reflected on, and discussed the shame experiences, and the perceived consequences of these. The interviews were audio-recorded and transcribed verbatim.

### Analysis

In all three groups, shame during clinical placement periods emerged as the dominant theme, without prompting from the interviewers.

Analysis was conducted as collaborative negotiations among all three authors. We applied *systematic text condensation* [24]: researchers a) read all material independently to obtain an overall impression and bracketing previous preconceptions; b) independently identified and grouped units of meaning, representing different aspects of participants’ experiences of shame-related events during their medical studies; c) condensed the contents of each of the coded groups; and d) critiqued and summarized the contents of each code group, aggregating the findings in three primary elements: the nature of participants’ shame-related experiences, learning environment factors that shaped medical students’ shame experiences, and the effects of shame experiences on the participants.

### Ethical issues

The Norwegian Data Protection Authority (NSD) granted approval for the study (ref 614041). The Regional Committee for Medical and Health Research Ethics found that the study did not fall within the mandate of the Norwegian Health Research Act and thus did not need to be appraised by the Committee.

The data was anonymized by the PI, and person-identifying information was removed from the transcripts. Only aggregated data and de-identified quotes, marked by group (G) and person (P) number, were utilized in this paper, ensuring that participants will not be identifiable on the basis of any personal information that they have provided.

## Results

All participants vividly remembered shame experiences across a range of clinical contexts, including rounds, clinical skills, patient visits, and assistance during surgeries and had rarely, if ever, shared these stories. As participants heard others’ examples of shame experiences, they disclosed their own narratives, expressing relief that such occurrences were common, and not a sign of personal inadequacy.

### Manifestations of shame

Students described shame as a threatened sense of self, characterized by perceptions of being fundamentally flawed, inadequate, unworthy, or deficient as a person. One participant said: *“You often feel like dirt under someone’s shoe”* (G3/P3).

The students highlighted a range of negative emotions associated with shame and embarrassment, including feeling angry, confused, desperate, frustrated, humiliated, insecure, intimidated, nervous, or sad. Shame reactions involved physical manifestations, such as moist hands, flushing face, pounding heart, queasy stomach, sweating, trembling voice, crying, or fainting. The somatic and emotional manifestations of shame were accompanied by negative cognitions.* “Every time I experience something (shameful), it goes on in my head forever” *(G1/P3).

### Social interactions contributing to shame reactions

The social situations that elicited shame fell into three main categories: clinician behaviour, suboptimal supervision, and disturbing interactions with patients and families.

#### Clinician behaviour

The participants described being newcomers in the clinical environments where they encountered personnel who were unprepared for, or hostile to, the presence of medical students, and whose avoidant or dismissive behaviour triggered shame when the students felt unacknowledged:* “There were constantly little hints that you’re insignificant, that you’re in the way and they don’t want you to be there” *(G1/P3).

Clinicians’ shame-inducing social behaviour consisted of breaches of social norms for respectful conduct, e.g., not acknowledging students’ presence, not making eye contact, not using their names or not shaking hands when meeting for the first time. Interactions that caused shame could be transient or more long-lasting and involve clinicians in larger groups, e.g., rounds. One student entered a physicians’ office at the start of a two-week hospital rotation:

I said, “Hi I’m a medical student.” This doctor turned around and looked at me and said “Congratulations!” Then he continued working. My heart was in my head, it was pounding, I was sweaty, and I thought “Sorry for existing”. (G2/P4)

Students felt devalued when doctors did not show up for teaching, were unaware that students were supposed to be with them at specified times, or avoided interactions all together. A female student who had identified her mentor, but not been greeted or acknowledged by him, shared the following experience:

He opened the door to this room, and it was a restroom. I was standing outside waiting for him to finish his business and he didn’t even bother to tell me that he was going to the restroom. I said, “fuck this, I’m going home.” I was like I have to go back tomorrow, and this is shameful. (G2/P4)

Students felt powerless and unable to voice protest regarding such shame experiences. The hierarchy of the clinic was frequently mentioned as a barrier to resistance.

A common theme in the groups was the shame caused by not being greeted:

If you haven’t said hi to the doctor and you haven’t said hi to the patient, then you are no one. You’re just a student, or a guy in a white coat. (G3/P2)

Students were often not addressed as a person, but as a generic “student”. They described this as a source of embarrassment, especially when patients were involved*. “Can the student leave the room”* (G1/P2).

#### Suboptimal supervision

The need to learn could become a source of bewilderment and shame when students were criticized or humiliated for lacking medical knowledge, for being inexperienced, or for being of little use in patient treatment.

Criticism without pedagogic support caused shame and loss of confidence: *“She commented on my performance in a very negative manner. I rarely feel ashamed or embarrassed, but I felt more and more insecure after that”* (G3/P4).

#### Disturbing interactions with patients and families

Meeting patients was not a source of shame, but the student-clinician-patient triad was consistently mentioned as a potentially shame-inducing configuration. Shame arose in students who witnessed unethical behaviour and felt they were unable to protest or protect the patient. In other examples, shame resulted from being linked with impolite or abusive clinicians:

I was in the emergency with this man with a mental disability. The doctor came to drain his abscess, he didn’t speak to me, he didn’t say hello, and didn’t speak to the patient. He just started to use the syringe, the patient was very afraid and was trying to get away from the doctor. I felt so ashamed about being associated with the doctor. (G1/P3)

Often shame emerged when supervisors involved students in patient interactions without providing introductions to the people who were present:

We walked into the room and they knew the doctor, but they didn’t know me. I hoped the family didn’t think “who is she, why is she here when our father has died”. I wished I had my Harry Potter invisibility coat. (G2/P3)

### Effects of shame on learning and professional development

Shame experiences had immediate or prolonged consequences that could impact negatively on students’ learning, career choice, or professional identity formation. Such effects included inability to focus on the task at hand, loss of motivation to learn or to practice, reluctance to request assistance, avoidance of certain situations or medical specialties, and loss of confidence in one’s ability to learn. Participants saw shaming as unhelpful for learning: *“It’s interesting, does shame make us better, or does it just kind of weigh us down? It made me practice more, but I still felt kind of useless” *(G3/P4).

#### Inability to focus on learning

Feelings of shame and attempts to defend against shame filled students’ awareness and made learning virtually impossible. “*In other situations when I feel shame I become really stressed and my mind is constantly trying to defend myself from feeling like nothing and then I’m not able to learn”* (G1/P3).

#### Loss of motivation

As a result of shaming experiences, students stated that they *“stopped engaging, listening and asking questions”*. In the following event, students are trying to understand the function of auscultation versus ultrasound in clinical assessment:

We said, “so what you’re saying is look at the whole picture and see all the pieces of the puzzle and see the whole patient”. And he said “no, no, you should just know what you’re doing”. He basically just said a big fuck you to all of us. For the rest of the hour, we did not ask any questions and we were just annoyed. (G3/P2)

#### Impact on career choice

Memories of a shameful interaction with a particular physician could deter students from pursuing a medical specialty. *“I think he was an internal medicine doctor and now he’s a geriatrician and I still take a mental two steps back every time I think about geriatrics and internal medicine” *(G1/P2).

#### Loss of confidence

Participants saw confidence as crucial for workplace learning to occur and reported that shame experiences typically impacted their ability to take initiative, to try things for the first time, and interact with patients in their role as medical student.

My professionalism and my ability to be a good med student and a good doctor are connected to confidence. This whole process gets thrown off if someone comes in and shakes that little confidence that you have. It’s much harder to launch yourself into something new if you don’t know if anyone is there to catch you. (G3/P4)

#### Influence on professional identity formation

Shame and loss of confidence threatened students’ integrity and professional identity development. The participants identified the medical hierarchy, and the need to belong and be like others in one’s social group, as potent mechanisms for change:

Should I respect my personality, who I want to be, or should I respect the hierarchy? And sometimes the struggle takes so much time and I miss the opportunity to be that good person I think I am. (G1/P3)

## Discussion

In this study, we sought to characterize the nature, contributing factors, and effects on learning and professional identity formation of shame in fifth- and sixth-year medical students during clinical placement periods. All our participants had experienced shame during interactions with clinicians, with intense negative self-evaluation and physical, emotional, and cognitive reactions causing anxiety, anger, withdrawal, loss of learning motivation, and avoidance of persons, specialties, or situations. These reactions were similar to those described in the psychology literature [1–3]. Our analysis also revealed important contextual and relational factors that provide insights into how shame is triggered in medical students during clerkship, and the effects shame may have on students’ skills learning, patient care, and professional identity formation. In this discussion we identify potential contributing mechanisms, outline implications of the findings, discuss limitations of our study, and indicate future developments in this important field of medical education research.

### The power of clinicians’ behaviour

While shame in young physicians has multiple origins, such as committing clinical errors, inability to relieve a patient’s suffering, remediation proceedings, or failing to reach personal career goals [10, 11], shame experiences of the medical students in our focus groups were invariably embedded in social situations within the work environment and were usually triggered by verbal or non-verbal clinician behaviour. Students do not have the formal responsibility of physicians, as their task is to learn, to discern, and comply with the norms of clinical medicine, to become real doctors [17]. However, as novices they are often not able to judge the adequacy of their own performance, and thus depend on the positive and negative verbal and non-verbal feedback they receive from clinician role models.

Our findings make clear that students are extremely vulnerable to social signals that may appear unimportant to clinicians themselves, such as not making eye contact and not using a student’s name. In her study of physician supervisors’ attitudes to intimidation in clinical teaching, Seabrook found that clinicians seemed to have “*limited awareness of how powerful and frightening they could seem to students*” [26].

### Possible causes of clinician insensitivity

Clinicians’ general blindness to their own impact on students does not explain the blatant dearth of courtesy, care, and professional ethics revealed in many of our participants’ examples. However, physician burnout, which is prevalent in hospital settings, is characterized by emotional exhaustion and depersonalization [27], and can be a source of insensitivity, lack of empathy, and negative attitudes. In addition, the “*impostor syndrome*” that can cause professional self-doubt in physicians at all career stages [28] may cause inquisitive students to appear threatening and elicit clinician avoidance or aggression. Similarly, feelings of personal inadequacy may make some students more prone to experiencing shame [11]. On a structural level, physicians have many incentives to see the clinic as a production system and, despite having a formal responsibility to teach and supervise, may perceive transient learners as impediments to efficiency. Medical students, especially beginners are, in anthropologist Mary Douglas’ words, “*matter out of place*”, or “*dirt*” [29, p. 36]. Douglas’ theory posits that a need for order in everyday life, as in a workplace, will inevitably make disturbances stand out as “dirt”. Our learners often felt like a burden, a bother, “like dirt under someone’s shoe”.

### Implications for medical learning environments

The power of shame to ruin self-confidence and motivation [10, 11] may shed light on the mechanisms at work in “*suboptimal learning environments*” (SoLE), where students get demoralized through interactions with staff, without being blatantly harassed [27]. Shame and associated defense mechanisms may also partly explain medical students’ loss of empathy [13] and withdrawal from patients [10]. Self-confidence and other-directedness develop in environments that provide psychological safety [11, 30, 31] and a sense of belonging [32]. The centrality of respect and care in clinical teaching is evident in the pedagogical ground rules of “experience-based learning”, which, based on a thorough review of the empirical literature, posits affective support as the most important contribution of clinician supervisors during clerkship [17, 18].

### Shame’s hidden workings

It may seem implausible that shame could be so central and yet hardly be mentioned in a vast literature on medical education. However, as has been articulated again and again in the sociological and psychological literature, shame is aversive and in itself shameful—“it is shameful and humiliating to admit that one has been shamed and humiliated” [2, 3, 32]. Shame is socially contagious, so that one person’s blushing causes unease in bystanders. This makes it unlikely that experiences of shame are inquired about or disclosed, as confirmed by our informants who had not shared their shame reactions with each other, despite the impact shame had on their lives and identities.

Yet the most important explanation of shame’s oblivion is its power to extinguish shame-provoking behaviour, as explored in the works of sociologist Erving Goffman on socialization and civilization [5]. Shame functions similarly to an electric fence, where previous experiences of pain make the cows stay well inside the “*correct*” field, never touching the wire, and blissfully avoiding negative feelings. Previous feelings of shame make people subconsciously monitor their future interactions for potential shame events, manoeuvring to avoid shame, as was illustrated by many of our participants’ examples [8].

Shaming of medical students is destructive, and difficult to detect and protect against, since the victims’ characteristic reactions to being shamed are self-loathing, aversion, and avoidance. Hence our finding that none of the students had ever voiced protest, and very few had shared shame experiences with peers.

Before any remediation is planned, one must understand that shame itself cannot be eliminated. It is, as explained above, a normal emotion that is constitutive of all social life by ensuring a degree of conformity and internalization of norms in any group. It is beyond the goals of this paper to suggest large-scale solutions to the problem of shaming in medical education. However, based on current knowledge, we believe that medical learning environments could be markedly improved by information sessions where students and faculty are given examples and basic facts about shame, and about the importance of emotion in human interactions in general, and are allowed to reflect on experiences that are not usually discussed [33]. Likewise, Douglas’ theory of “*dirt*” could shed light on mechanisms that operate on the systems level, influencing how students are perceived in a work environment geared for production.

### Limitations

The findings of this qualitative study are based on a small number of focus groups that may not fully capture all shame experiences, though all three focus groups came up with similar experiences and topics. Individual interviews may provide an opportunity for discussion of shame events that students were not comfortable discussing in a group format.

Findings are based on a limited number of students at one university in Norway and do not in themselves reveal how common it is for clinicians to shame medical students, or for students to experience shame. The content of students’ recall may have been influenced by the interactions between researchers and participants. Shame is a highly complex phenomenon, and further qualitative research is needed to explore how the social characteristics and pedagogical qualities of different work environments incite or prevent shame in student-clinician interactions. Quantitative inquiry, such as survey-based studies, may document the variations and prevalence of shame in clerkship and its impact on various forms of learning.

## Conclusion

In this study we found a wide range of shame-related experiences during clerkship, triggered by clinician supervisors’ breaches of trust, courtesy, or professionalism. Shame caused avoidance behaviour and harmed students’ confidence, motivation, learning, and professional identity development. Our findings suggest that shame may be particularly important for understanding how student mistreatment arises and what its effects are on learning and health—and may also suggest why remediation and improvement of medical learning environments have proven so difficult [34]. The aversive nature of shame makes it difficult to talk about, yet awareness, transparency, and reflection are needed. It is time to change medicine’s traditional inattention to the workings of emotion in health, sickness, work, and learning.
